# Errors in Spectrophotometry and Calibration Procedures to Avoid Them

**DOI:** 10.6028/jres.080A.060

**Published:** 1976-08-01

**Authors:** A. G. Reule

**Affiliations:** Carl Zeiss, 7082 Oberkochen, Germany

**Keywords:** Bandwidth, calibration, errors in spectrophotometry, interferences, multiple reflections, photometric linearity, polarization, sample characteristics, stray light, wavelength accuracy

## Abstract

Based on simple principles, spectrophotometry nevertheless demands a lot of precautions to avoid errors. The following properties of spectrophotometers will be discussed together with methods to test them:

Spectral properties—wavelength accuracy, bandwidth, stray light; photometric linearity; interactions between sample and instrument—multiple reflections, polarization, divergence, sample wedge, sample tilt, optical path length (refractive index), interferences.

Calibration of master instruments is feasible only by complicated procedures. With such a master instrument standards may be calibrated which greatly simplify performance checks of instruments used for practical work. For testing high quality spectrophotometers the use of emission lines and nearly neutral absorbing solid filters as standards seems to be superior, for some kinds of routine instruments the use of absorption bands and liquid filters may be necessary.

## I. Introduction

The comparison of measured results of different optical parameters reveals considerable differences in accuracy. There is no difficulty in stating refractive indices to within 5 decimals and tables of line spectra quote wavelengths to within 6 to 7 significant digits. In contrast, the transmittance of an object can be indicated to within 0.1 percent only, and even this allows merely statements as to random but not to systematic errors.

In 1973 the College of American Pathologists carried out a comparative test. A number of solutions was sent out and measured with spectrophotometers in 132 different laboratories. The results showed coefficients of variation in absorbance of up to 22 percent. When the test was repeated in 1974, the coefficients of variation among 135 laboratories still amounted to maximally 15 percent; although 24 laboratories had been excluded by a special test because their photometers had more than 1 percent stray light at 240 nm. This corresponds to a coefficient of variation in transmittance of up to 11 percent (table I) [l].^1^

It is thus not surprising that spectrophotometer users call for standards to test their instruments.

These tests and many similar ones have been and are still carried out with solutions having several wide transmission maxima and minima. As references for photometric *and* wavelength accuracy of the instruments, measuring wavelengths are sometimes stipulated for the extremes and for values on the slopes of the transmission curves, although such an inference is often impossible. The author would therefore suggest separate tests for wavelength accuracy and photometric accuracy, even for routine work. Only then will it be possible to prove by a test not only the unreliability of measurements, but also to derive quantitative parameters suitable for correction of the results. The individual sources of error are, of course, dealt with and eliminated separately if instruments and equipment are tested in laboratories issuing and calibrating standards.

The individual sources of error and possible test methods are discussed in the following, with special consideration of two points of view: fundamental tests for specific errors in the standards laboratories and tests by the average users of spectrophotometers in clinical or analytical laboratories.

The result of a spectrophotometric measurement is expressed by two numbers : wavelength and transmittance. The transmittance of a sample as a function of the wavelength is generally expressed by a curve. Since the transmittance may vary more or less strongly with wavelength, both the spectral characteristics and the accuracy of the photometric result must be considered. Three groups of characteristics are therefore discussed: spectral characteristics, genuine photometric characteristics, and optical interactions between sample and photometer.

In practice there are other sources of error, such as environmental effects on photometer and sample, temperature, line voltage fluctuations, vibrations, contamination, or heating of the sample by the photometer. All these factors may impair the measured result, and ways and means are known to test and eliminate them. They are therefore disregarded in the following discussion.

There are numerous publications about tests of this kind, and each individual problem has been discussed in the relevant literature. This paper gives a survey of the problems and discusses methods the author would like to recommend or to warn against. The study is limited to photometers for UV, VIS, and near IR. A good survey of the literature published until 1972, especially regarding tests for photometric linearity, is included in the proceedings of the Conference on Accuracy in Spectrophotometry and Luminescence Measurements, held at the National Bureau of Standards in March 1972 [2], particularly in the contributions of Clarke [3], Mavrodineanu [4], and Sanders [5].

## II. Testing the Spectral Characteristics

### A. Accuracy of the Wavelength Scale

**Figure 1:**
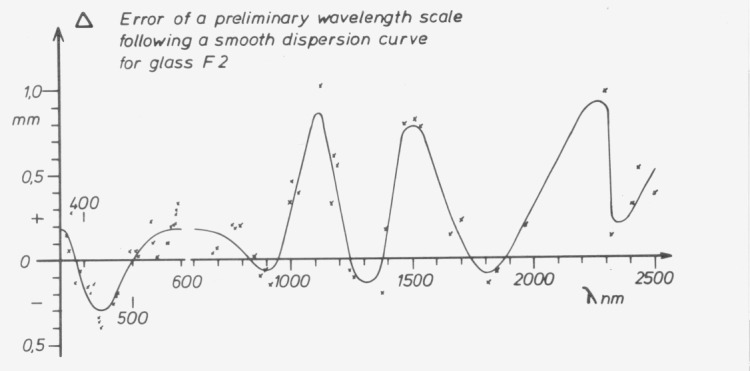
Irregularities in the dispersion of a prism made of F–2 glass (Schott). Shown are differences between actual transmitted wavelengths and readings of a preliminary wavelength scale following a smooth dispersion curve. Projection scale with 50× magnification see figure 2.1 mm on the groundglass screen corresponds to about 0.038 nm near 400 nm and about 5.5 nm near 1500 nm.

**Figure 2:**
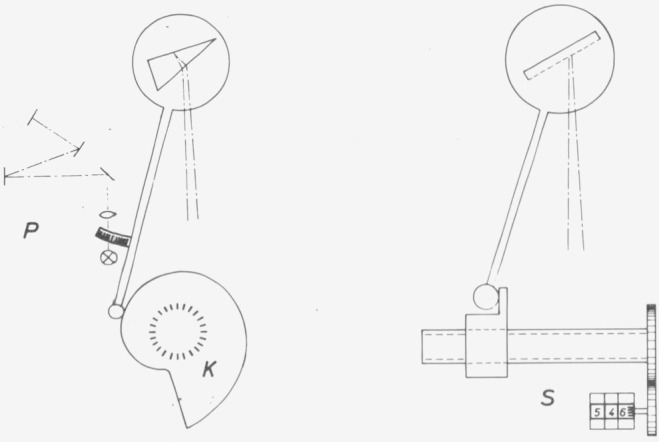
Different wavelength display systems. To the left: (a) (Micro) projection scale rigidly connected with prism, (b) Wavelength cam and scale fixed to cam. To the right: (c) Sine bar mechanism,
nonlinear, high uniformity of different instruments of the same type, no backlash, no wear, no parallaxcan be made linear in λ or *v* less uniform, subject to backlash, wear and parallax (a projection system in connection with a cam avoids parallax only)linear in λ, uniform, subject to backlash and, less pronounced, to wear and parallax. Periodic errors possible (see fig. 3).

**Figure 3:**
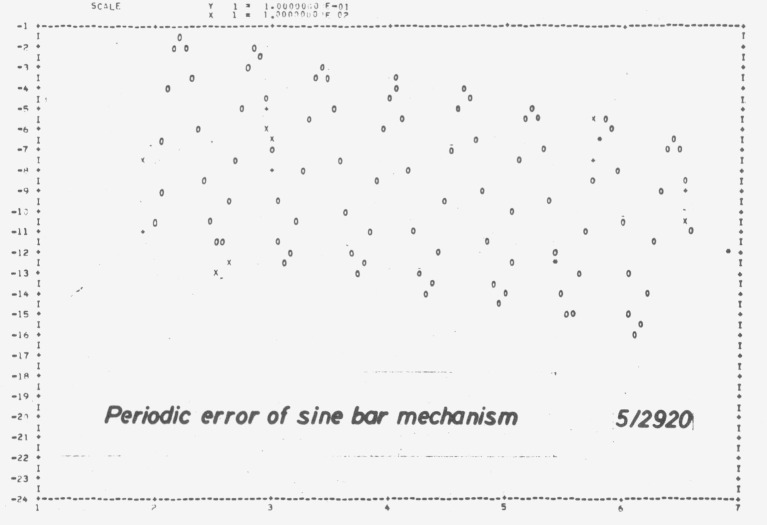
Periodic error of a sine bar mechanism (in this case due to an unsuitable ball bearing). x measured lines (Hg), + and 0 best fitting curve.

The wavelengths of a great number of emission lines within the ultraviolet and visible spectral regions are known exactly. Standards or industrial laboratories which have to supply the data for such scales encounter no difficulties in testing wavelength scales. Two facts deserve special mention:

- Even in regions without absorption the dispersion of prism materials is not as homogeneous as may be expected. Thorough tests therefore require measurements at a great number of wavelengths (fig. 1).
- The fundamental errors of a sine bar mechanism (fig. 2), which is commonly used for rotation of gratings, should be covered by checks at four wavelengths. This does not apply, however, to periodic errors of the lead screw or its bearing, which sometimes cause surprising discrepancies (fig. 3).

A single wavelength suffices to test an instrument supplied free of defects by the manufacturer, because it is unlikely that the tested wavelength remains unchanged in case of damage during shipment or other sources of error. This is not true, however, if the user or the manufacturer’s maintenance service carries out adjustments, which generally requires the checking of two to three wavelengths. This is not necessary in case of special designs (fig. 2a).

**Figure 4:**
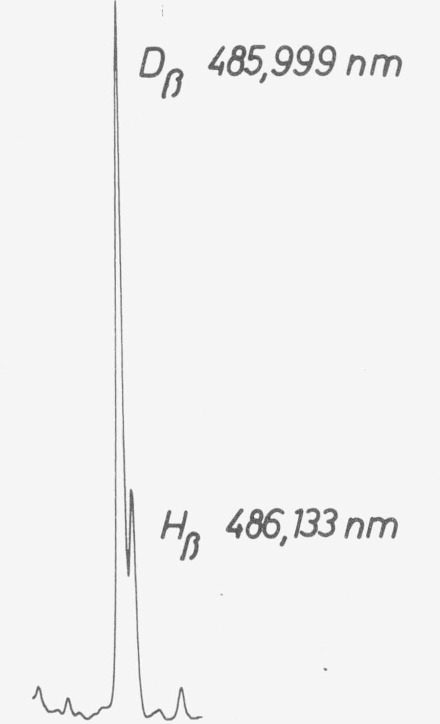
Line emission of a commercial deuterium source showing *D* and *H* lines. The H/D intensity ratio is likely to increase with age.

If the user wants to check the wavelength in instruments equipped with a deuterium source, he should use the emission lines of deuterium. Note the differences between deuterium and hydrogen lines (table II). However, many light sources contain in addition to deuterium higher or lesser amounts of hydrogen, which cause errors if the resolving power of the instrument is not adequate to separate the lines (fig. 4).

In an instrument without a deuterium source the aforementioned average user cannot be expected to equip it with a line source to check the wavelength ; therefore absorption bands must be used for the test. The bands of the well-known didymium glass are not suited for wavelength checking because they are too wide, temperature-dependent and because didymium glass may contain varying amounts of several absorbing rare earths.

**Figure 5:**
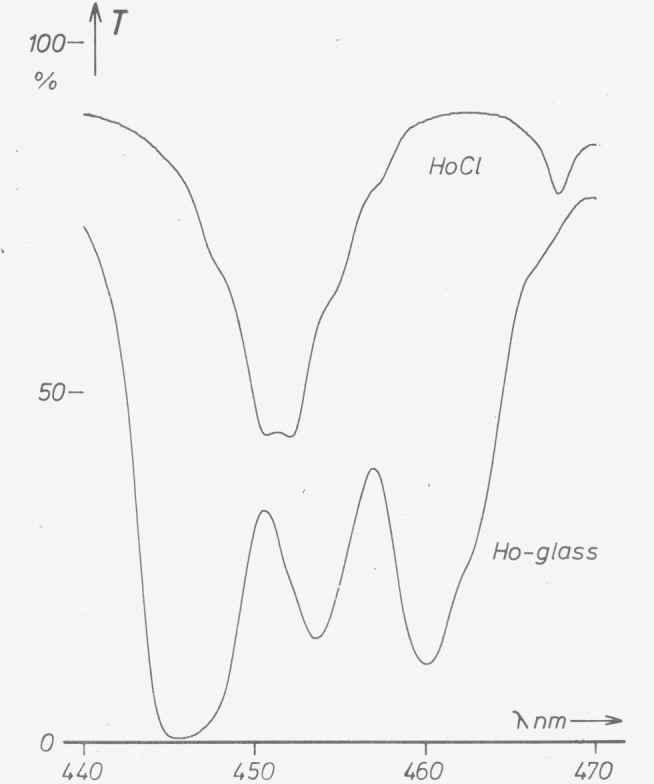
Spectral transmittance of Holmium in aqueous solution (1.14 g *Ho_2_O_3_* in 100 ml 0.2 *N HCl*; 1 cm) and as an absorbing constituent of glass. Part with the greatest differences; minor differences are observed with the other bands.

Holmium in aqueous solution has rather sharp absorption bands. Most of the bands of holmium glass are at nearly the same wavelengths as for holmium solutions but are somewhat wider with one striking difference near 450 nm (fig. 5). Because the glass matrix seems to influence Ho absorption there arises the question whether the wavelengths of absorption maxima of Ho-glass are the same for all melts.

**Figure 6:**
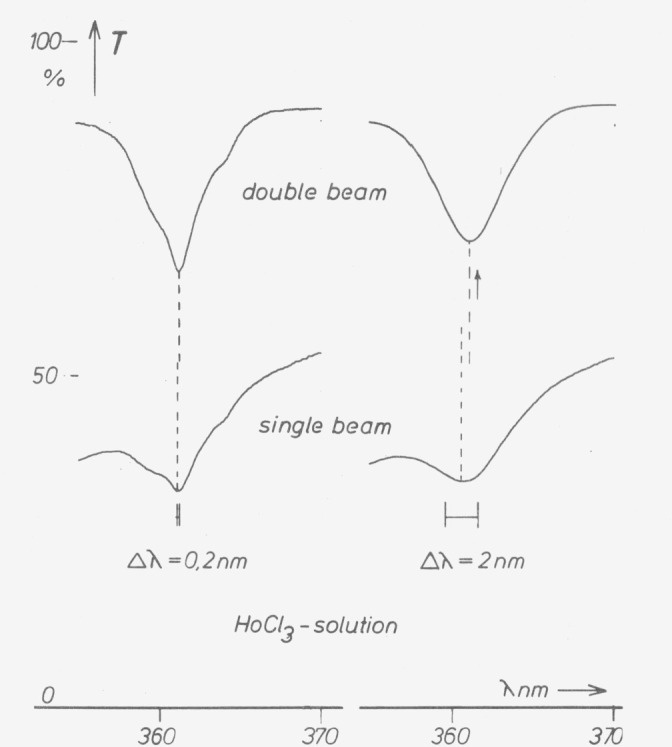
Spectral transmittance of Holmium chloride solution as in figure 5 measured in a double-beam instrument (ZEISS DMR 10) at 0.2 nm (left) and 2 nm (right) bandwidth (curves above), and in single-beam mode (curves below). In single-beam mode the error is less than 0.05 nm at 0.2 nm bandwidth. At 2 nm bandwidth the double-beam curve shows a shift of 0.1 nm of the minimim due to skewness of the true transmittance. The single-beam minimum is shifted by 0.15 nm from the double-beam minimum (0.25 nm from the true one) due to skewness of the empty instrument signal.

**Figure 7:**
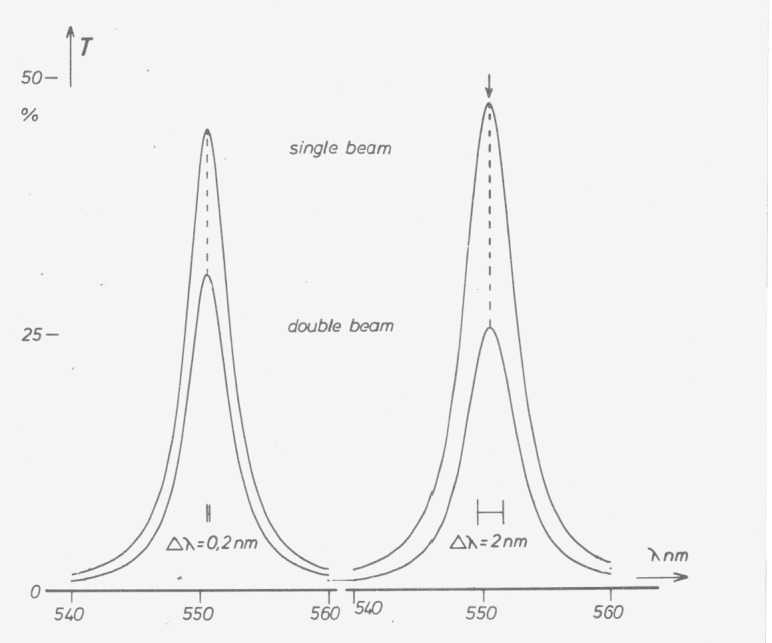
Spectral transmittance of an interference fitter for wavelength calibration measured at 0.2 nm and 2 nm bandwidth in double-beam mode (curves below) and single-beam mode (curves above). There is no difference in the position of the maxima up to 2 nm bandwidth; 0.4 nm shortwave shift at 5 nm.

In single-beam instruments only narrow bands measured with small bandwidth allow the determination of absorption maxima by simply scanning the wavelength. If the bands or bandwidths are wider, the positions of the maxima are shifted due to the spectral variation of signal in the empty instrument (fig. 6). This can be avoided by using an absorption or transmission maximum near the maximum of the instruments’ response curve, which is achievable by special interference filters (fig. 7). We found such interference filters to be most helpful for wavelength checks of instruments with bandwidths between 2 and 10 nm. The filters must, of course, be most carefully produced, and the wavelength of maximum transmittance given for each individual filter.

### B. Bandwidth

**Figure 8:**
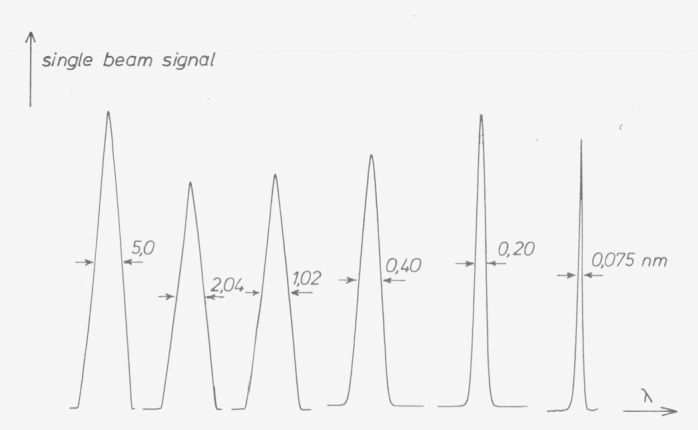
Bandwidth determination with isolated emission lines. Recorded in single-beam mode with ZEISS DMR 10 Recording Spectrophotometer and mercury line 546.07 nm. Abscissa scale expansion changed to give nearly equal widths of the four larger bandwidths; ordinate expansion, voltage adjustment and neutral filters used to make amplitudes nearly equal. Nominal bandwidths (“spectral slit widths”) set to 5-2-1-0.4-0.2 and below 0.1 nm.

Bandwidth is best checked by recording the signal as a function of wavelength, when the monochromator is irradiated with an isolated emission line (fig. 8). Bandwidths well above the limit of the resolving power produce the well known triangle, whereas bandwidths approaching the limit of resolving power result in a rounded curve.

The aforementioned deuterium lines are less suited for the determination of the bandwidth, because of other lines and continuous background in their vicinity.

The dip between two emission lines can be easily interpreted only if both lines are of equal intensity and if the transmission profile of the monochromator is symmetrical.

Absorption bands are generally not sufficiently pronounced or isolated. The only definite conclusion that can be derived from the resolution of two bands is that the bandwidth is smaller than the distance between the two.

As far as the author knows, previous comparative tests did not include bandwidth checks by the user. There is little danger of corresponding changes in good instruments. If a check by the user is necessary, the separation of absorption bands is probably the most convenient procedure.

### C. Stray Light

In this connection the term stray light refers to heterochromatic stray light; that is, light of wavelengths outside the bandpass of the monochromator. Due to scattering, a fraction of this light passes through the monochromator, while outside the monochromator this light follows the regular path. In Germany the expression “Falschlicht” is used, similar to “false light,” sometimes used in the U.S., to distinguish this unwanted radiation from radiation scattered by the sample or sample compartment optics and deviating from the regular path. The fraction of the signal (electric current) which is due to stray light falling on the detector is important for the measurement. This is called the stray light ratio and is especially large at the ends of the spectral range of the instrument, where slit width or amplification must be large. However, with a single monochromator this stray light ratio does *not* change as the slit width or the amplification changes [6].

There are various methods to determine the stray light ratio, of which the different versions of the absorption method, the slit height method, and Preston’s method are of practical relevance.

The absorption method requires a substance that is completely opaque within the region of two bandwidths and is completely transparent outside this region. If the stray light occurs exclusively at the longwave or shortwave side, like at the ends of the spectral range, the substance may only have a longwave or shortwave cutoff.

**Figure 9:**
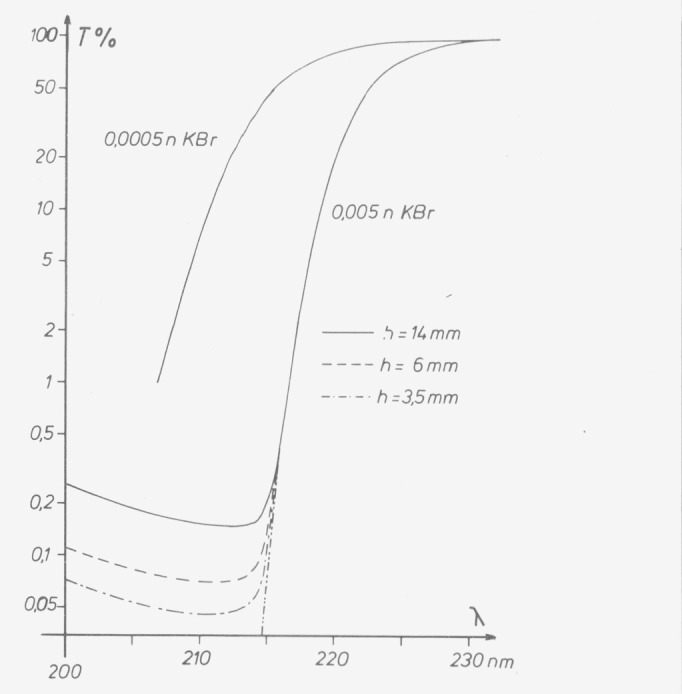
Determination of stray-light ratio and its variation with the slit height using *KBr* solutions in 1 cm cells. The true transmittance of 0.005 N solution (dotted line) is calculated from the transmittance of 0.0005 N solution.

There is no substance which provides an ideal step function transmittance at one wavelength, but there are a number of substances having sharp cutoffs or narrow absorption bands in the center of the spectral range. Sharp shortwave cutoffs can, for instance, be produced with chlorides, bromides, and iodides (fig. 9). A 15 g/1 aqueous solution of sodium chloride has, for instance, at 200 nm and 1 cm path length a transmittance of less than 0.1 percent. The cutoff is temperature-dependent, and even at low temperatures the concentration fulfills the requirements well. Generally the solution is selected as follows: a solution with suitable cutoff is measured in low concentration so that the transmittance lies between 20 and 50 percent (there is only little influence of stray-light) and the required concentration or path length is calculated. If the aforementioned sodium chloride solution is, for instance, used to test a double monochromator, 2 cm instead of 1 cm path length are used.

There are no substances with similarly steep longwave absorption cutoffs, but interference barrier filters are of some help. The true transmittance of each filter must, of course, be measured in a double monochromator. The instrument user may carry out checks with absorbing solutions and interference barrier filters.

Slit height variation is the second method for stray-light tests [6]. Contrary to its independence of slit width, the stray-light ratio decreases linearly with the slit height, provided the heights of the entrance and exit slits are reduced simultaneously. For a quantitative determination all parts of the slit must contribute uniformly to radiant flux and signal. This requirement is hardly ever met, and reduction of the slit height therefore only reduces the stray-light ratio without allowing its quantitative determination. Some manufacturers equip their instruments with facilities to change the slit height, thus offering the user the possibility of estimating and reducing the stray light (fig. 9).

In Preston’s method [7] one half each of the entrance and exit slits is covered. If the covering is such that an image of the free part of the entrance slit is produced on the free part of the exit slit, the transmitted radiation still contains signal light and stray light. Owing to the reduced slit height the stray light ratio is about fifty percent less than originally. If the other half of the exit slit is covered, only stray-light leaves the monochromator, which can be quantitatively determined. The accuracy of the determination is impaired, though, because for safety reasons the covering must be larger than the part of the slit through which the signal light passes. As this method requires manipulations in the slit plane, it usually cannot be applied by the instrument user.

## III. Testing Linearity

### A. Representation of Linearity Errors

**Figure 10:**
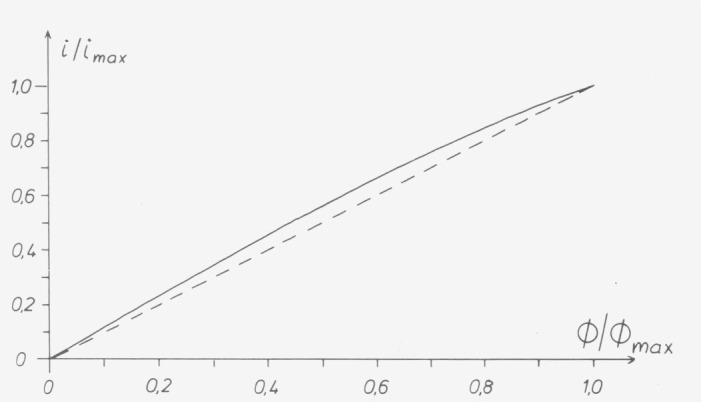
Nonlinearity of detector response (vertical distance between the characteristic curve giving the current i as a function of the incident radiant power ϕ and the straight line).

Two methods of representation are in use which are both based on the fact that the photocurrent *i*_max_ (or the equivalent readout on an instrument) which corresponds to a maximum value Φ_max_ of the radiant flux is stipulated as a reference point. If *i_d_* is the dark current, the following equation should hold for a current i_lln_ which has a strict linear relationship with the radiant flux Φ (fig. 10):

$$(i_{\text{lin}}-i_{d})/(i_{\max}-i_{d})=\phi /\phi_{\max}.$$ (1)

Differences between the actually measured value *i* and the value i_lln_ derived from the equation are called linearity errors:

$$\Delta i=i-i_{\text{lin}}.$$ (2)

**Figure 11:**
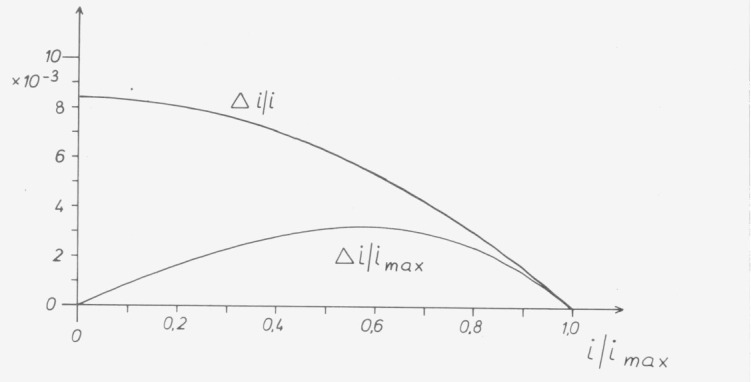
Expression of the nonlinearity by *Δi/i _max_* or *Δi/i*.

Both methods express the linearity error as a fraction of a measured result, usually in percent. The two methods differ in that the error is referred either to the maximum value *i*_max_ or to the actual reading *i* (fig. 11). In both cases the determined errors or derived corrections are valid only for measurements which are based on the radiant flux Φ_max_. Which method is chosen depends on the user. In practice the reference to the maximum value is somewhat easier for the correction of transmittance, because the correction term can be added directly. Reference to the individual value has advantages if the transmittance is low or for measurements of absorbance A, because the following equation holds:

ΔA=0.434(Δi/i). (3)

### B. Photomultiplier Characteristics

Most precision spectrophotometers are equipped with photomultipliers as detectors. Their characteristics; such as, spectral sensitivity of the cathode, dependance of gain on voltage, voltage drop at the anode resistor, variations of sensitivity across the cathode or with direction of incidence etc. are so well known that they will not be discussed here.

We should like to mention just a few effects which are not so well known. The first is the temperature-dependent sensitivity of the cathode [8] (table III). If a radiant flux of 10 nW falls on a cathode with a metal substrate, the rate of temperature increase due to absorption of radiation is approx. 0.5 × 10^−6^ °C/s. This value is so small that even with irradiation over extended periods the sensitivity does not change markedly due to the temperature increase caused by the radiation. However, the temperature rise time of cathodes on glass or quartz substrates (as in end-on types) may reach values which are interfering, yet are still too slow to reach thermal equilibrium.

**Figure 12:**
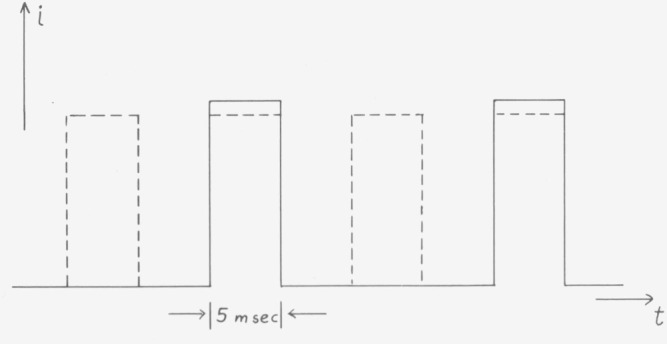
Short-term memory effect of photomultipliers. The multiplier is at first illuminated with light pulses which result in the current given by the solid lines. If equal light pulses (dashed lines) are interposed between the first ones, the current amplitude is reduced by an amount which depends on multiplier type and specimen. With pulses giving a current amplitude of 10^−7^ Λ a reduction of −1.3 percent was observed, which took about 5 s to reach the new level.

Another troublesome characteristic of detectors is that the current generated depends not only on the present but also on the previous irradiation, an effect which is known as memory effect. Most detectors have memory effects of some tenths of a percent for a duration of a few seconds, if they are covered for a few seconds after long exposure to light. This holds true even when the anode currents are kept to values between 10^−8^ to 10^−7^ A. Some detectors show much larger memory effects. The effect is not only noticeable upon irradiation with continuous light, but also if the light is chopped at 50 Hz. Only recently, we observed a change of the signal amplitude in light pulses of 5 ms width and 15 ms dark time, dependent on whether or not pulses of equal amplitude and width were interspaced between these pulses. The change in amplitude of the original pulses amounted to −1.3 percent and covered a period of approximately 5 s (fig. 12) [9].

In keeping the measuring accuracy below 10^−4^ of the maximum current, there is the danger that these memory effects affect not only the measurement of the sample under test but also the methods for linearity tests. It will be difficult to distinguish between memory effect and nonlinearity. All readings must be done in a sequence of time which is exactly stipulated and reproducible. Residual memory effects in the results may be recognized by changing the time between readings.

### C. Light Addition Methods

**Figure 13:**
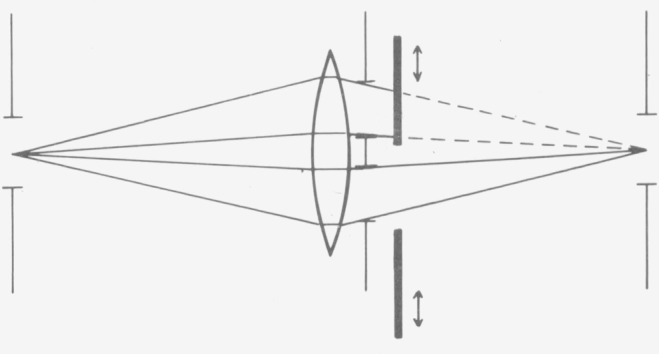
Arrangement for the double-aperture method.

**Figure 14:**
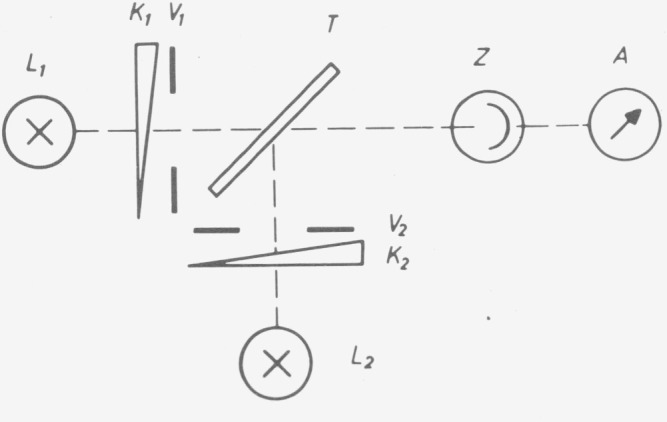
Arrangement for the supplementary-light method.

As indicated elsewhere [10, 11], there are no samples the transmittances of which are known with sufficient reliability without photometric measurement. The standards laboratories must therefore determine the linearity of the instruments by special test methods. The addition method is the basic procedure for the design of an equidistant scale of any measuring parameter. Different versions of the method are in use in photometry [12]. There are setups with several apertures and setups with several light sources. The setups with two apertures (fig. 13) [13, 3, 4], with many apertures [14, 5], and with two light sources (fig. 14) [15, 10] are especially important in practice (the first—to the author’s knowledge—and one or two recent papers are cited respectively).

Setups with two or several apertures have the advantage of needing only one light source, that is the one in the instrument under test. Neither is additional optics required in the beam path, so that the state of polarization of the radiation remains unaffected. However, the light in the instrument is reduced considerably by the aperture diaphragms, so that the method can only be applied if there is sufficient light available and if the cross section of the light beam is large. For these reasons, these methods cannot be applied to commercial instruments with small beam cross sections or other beam geometries (e.g., variable apertures, rotating sectors and mirrors, modulators etc.) that would be perturbed by the insertion of additional apertures. For example, an existing variable aperture stop may cause incomplete illumination of an inserted double aperture arrangement and, thus, render it useless. If, as another example, in an instrument using chopped light, the shape of the intensity versus time (as can be observed by an oscilloscope) is changed by an inserted double aperture other than merely in amplitude, linearity cannot be tested this way. Methods using two or several apertures however are well suited for testing the linearity of special equipment the design of which allows the use of the aperture method.

To guide the light beam the method with two sources requires more optical parts and a semi-transparent plate which may change the state of polarization. However this method can be adapted to commercial instruments as demonstrated by the author, even in a double-beam instrument [10].

### D. Other Methods

As mentioned before [10, 11, 16], tests based on Lambert’s or Beer’s laws alone are inadequate. At least one point of the photometer scale must be established in another way. The same is true of the method discussed in detail in reference [11], in which neutral density filters or perforated screens are measured for varying attenuations of the beam.

**Figure 15:**
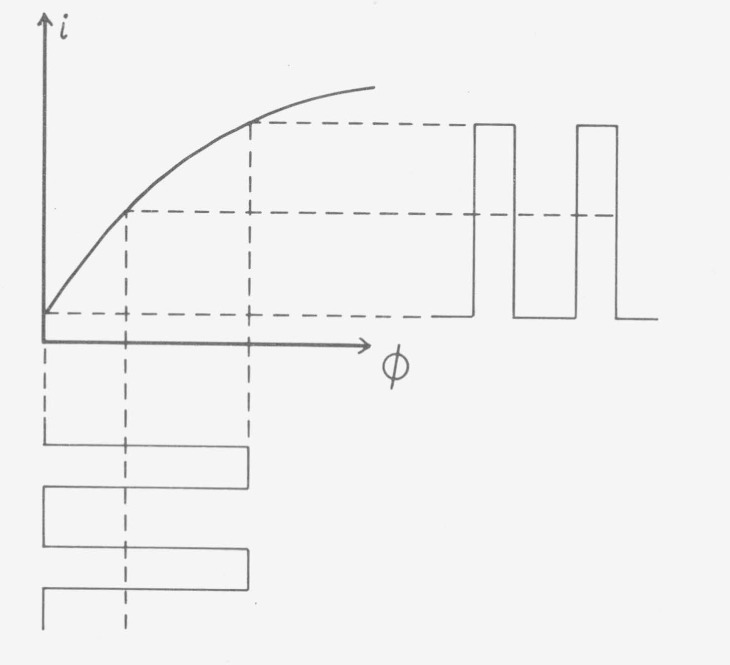
Current from a nonlinear fast detector illuminated through a rotating sector. With the sector set to 40 percent open time the light pulses (shown below the dectector characteristics) will cause current pulses with 40 percent on time (shown to the right of the detector characteristics), i.e., strict linearity. A sample of 40 percent transmittance illuminated with continuous light will cause a current amounting to about 50 percent of that without sample and showing striking nonlinearity.

Rotating sectors are not suited to test photometer scales of equipment with detectors having response times which are short compared with the open and dark periods of the sector [5, 10]. The detector signal changes between the dark period and the full value; the part of the characteristic curve which is essential for the measurement of an absorbing sample is not passed (fig. 15). The test can be made with a rotating sector only if the open and dark periods of the sector are short compared with the response time of the detector (in the IR range, for instance).

Inspired by a theoretical treatment of Hansen, who introduced the derivative of flux with respect to reading [17], Ploke [18] has applied a simple method to test the linearity of detectors. A weak, chopped radiation is supplied to the detector under test, and is measured with a lock-in amplifier. Additional unchopped light is then supplied to the detector and the signal of the lock-in amplifier is observed, which approximates the derivative of reading with respect to flux. Each change of the signal is interpreted as a curvature of the characteristic curve and thus as a nonlinearity. This holds true if there is no memory effect and if the characteristic curve is the same for continuous and chopped radiation. In other cases the distinction of nonlinearity, frequency dependence and memory effect becomes difficult. The same disadvantage applies to the following method.

Jung [19] states an interesting method to determine the nonlinearity from the beat frequency of two radiations chopped at different frequencies. Although this method is interesting for testing multipliers, it is not suited for the direct calibration of a photometer. Jung also describes a method to improve the linearity of multipliers [20], which uses chopped measuring light and adds unchopped light to the weaker of the two light portions until the mean photocurrents are equal in both cases. The amount of added unchopped light need not be measured. However, Jung’s own theoretical treatment of the method reveals that only the nonlinearity which is proportional to the radiant flux is rendered completely ineffective. Higher-order components of nonlinearity are reduced, but not eliminated. For this reason Jung’s method can be assumed to improve linearity, although it does not eliminate the necessity to measure it.

The difficult testing of photometer linearity can only be carried out in standards laboratories. They must provide standards of known transmittance which are then applied by the user to test his photometers. The interaction between sample and photometer must be considered for the calibration of the standards and for their use.

## IV. Interaction Between Photometer and Sample

### A. Definition of True Transmittance

If the transmittance of a specific sample is measured in several photometers with defined linearity or known nonlinearity and arithmetically corrected, this would not be a guarantee for consistent results. This is due to a number of interactions between sample and photometer. The values obtained by measurements in a linear photometer cannot be regarded as true transmittance.

**Figure 16:**
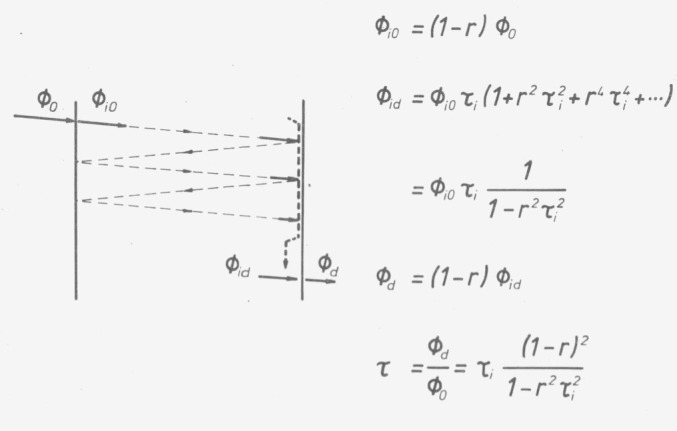
Influence of surf ace reflections on the transmittance of a homogeneous, isotropic, optically clear sample with plane parallel surfaces passed by a nearly parallel beam of light.

Corresponding to international standards organizations, we start with the theoretical definition of the internal transmittance of a homogeneous sample limited by parallel plane surfaces. This internal transmittance *τ_i_* is defined as the ratio of the outgoing radiant flux at the inside of the exit surface to the incoming radiant flux at the inside of the entrance surface. The radiation is assumed to be a quasi-parallel, sufficiently monochromatic light beam perpendicular to the boundary surface of the sample (fig. 16). A reflectance of the radiant flux of the amount *r* is assumed, owing to the refractive index discontinuity at the boundary surface. It follows that the transmittance determinable from the outside is, for a single passage of the radiation,

$$\tau =\tau_{i}{\left(1-r\right)}^{2}.$$ (4)

The radiant flux reflected by the inner exit surface returns to the entrance surface where it is again partially reflected. The result of the infinite series of multiple reflections is a somewhat higher transmittance.

$$\tau =\tau_{i}\frac{{\left(1-r\right)}^{2}}{1-{\tau_{i}}^{2}r^{2}}.$$ (5)

The first question is to what extent these multiple reflections will become effective in the photometer. Even in photometers with a collimated beam the light is not exactly parallel. The more it is reflected the more it diffuses and finally does not reach the detector any more. Therefore, in practice, an infinite number of reflections need not be considered; the first back and forth path in the sample alone produces the transmittance

$$\tau =\tau_{i}{\left(1-r\right)}^{2}(1+{\tau_{i}}^{2}r^{2}).$$ (6)

For normal glass, *r* is approx. 0.04 (4%). Although the first back and forth reflection will change the transmittance of an absorption-free sample (*τ_i_*=l) by about 0.16 percent, the following would result in only 2.5×10^−4^ percent and can be neglected. The thickness of absorbing glasses used as standards is typically about 2 mm. Even in photometers with focused beam the expansion of the light beam over this distance is not large enough so that an appreciable part of the reflected light would be lost. Thus, it is justified to define the transmittance according to the eq (6). After elimination of the causes of error mentioned, it can be expected that photometers with correct display will also measure this value, which does not differ from (5) within the stipulated measuring accuracy. The flux reflectance *r* was used for the calculation; thus possible interferences of coherent light are not considered for the definition. They will be disucussed further.

### B. Obliquity Effects

Not all rays passing through the sample in a photometer are parallel. This applies not only to commercial photometers with focused beams, but also to photometers with a collimated beam. The aperture limiting the divergence must have a finite diameter if energy is to pass. Deviation of the rays from the normal on the sample surface causes path length and reflection errors.

**Figure 17:**
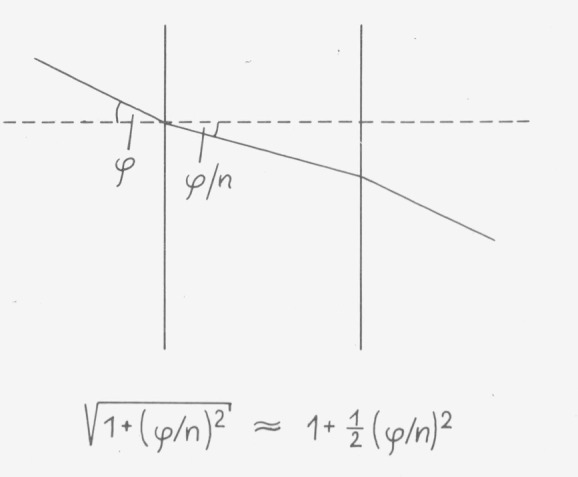
Path length error caused by oblique incidence.

If the optic axis is perpendicular to the surface and if the path length for the maximum external angle of incidence *φ*_max_ does not exceed (1 + *∊*) of the path length for normal incidence (fig. 17) the following equation must hold

$$\varphi_{\max}^{2}≦2n^{2}\epsilon$$ (7)

where *η* is the refractive index of the sample.

If the light beam at the sample has a cross section A, this determines the light gathering power. From this value, the spectral radiance *L*_λ_ of the light source and the bandwidth Δλ used for measurement, the radiant flux, and finally the number *N_t_* of photoelectrons per unit time at the cathode can be calculated as follows

$$N_{t}=L_{\lambda }\cdot \Delta \lambda \cdot A\cdot \pi \cdot \varphi_{\max}^{2}\cdot \tau \cdot q/hv$$ (8)

where *q* is the quantum yield of the cathode, *τ* the transmittance of the entire optical system including the efficiency of a sphere (if any), *h* Planck’s constant, and *ν* the frequency of light. Due to shot noise, the relative precision for a measurement with an integrating period *t* is

$$\frac{\Delta N}{N}=\frac{1}{\sqrt{N}}=\frac{1}{\varphi_{\max}}\cdot \frac{\sqrt{h\cdot v}}{\sqrt{\pi \cdot L_{\lambda }\cdot \Delta \lambda \cdot A\cdot q}\cdot \sqrt{\tau \cdot t}}$$ (9)

Numerical values are given in table IV. As can be seen from the table, difficulties are to be expected only if one tries to limit errors substantially below 10^−4^.

The obliquity is generally greater in commercial photometers. The photometer reads an average value ${\bar{\tau }}_{i}$. It must be considered, however, that the instrument does not average the path lengths or absorbances, but transmittances. For a sample with an internal transmittance *τ_i_*_0_ for normal incidence, if traversed by a light beam containing rays of obliquity *φ* with a weight factor *g*(*φ*) the following equation holds

$${\bar{\tau }}_{i}=\int g(\varphi )e^{\left(\text{ln}\;\tau_{i0})/\cos\;(\varphi /n\right)}\cdot d\varphi .$$ (10)

Developing both the cosine and the exponential function in power series and truncating after the first term, yields the following result

$${\bar{\tau }}_{i}=\tau_{i0}\left[1-\ln\;\left(1/\tau_{i0}\right)\cdot \frac{1}{2n^{2}}\int g(\varphi )\cdot \varphi^{2}\cdot d\varphi \right].$$ (11)

When measuring samples which attenuate due to absorption (not reflection) such a photometer seems to read a linearity error referred to the maximum value of

$$\Delta_{\tau_{i}}=-\frac{1}{2n^{2}}\cdot \tau_{i0}\cdot \ln\;(1/\tau_{i0})\int g(\varphi )\cdot \varphi^{2}\cdot d\varphi$$ (12)

or a linearity error referred to the corresponding individual value of

$$\Delta \tau_{i}/\tau_{i}=-\frac{1}{2n^{2}}\cdot \ln\;(1/\tau_{i0})\int g(\varphi )\cdot \varphi^{2}\cdot d\varphi .$$ (13)

If *g* (*φ*) contains all beams within a circular cone with the maximum aperture *φ*_max_ (corresponding to the geometry of a point source irradiating a circular aperture, or corresponding to collimated light if the aperture limiting the divergence appears under the aperture angle *φ*_max_ seen from the lens) the function *g*(*φ*) with due consideration of the normalizing condition

∫g(φ)⋅dφ=1 (14)

has the form

$$g(\varphi )=2\varphi /\varphi_{\max}^{2}$$ (15)

so that

$$\int g(\varphi )\cdot \varphi^{2}\cdot d\varphi =\varphi_{\max}^{2}/2$$ (16)

**Figure 18:**
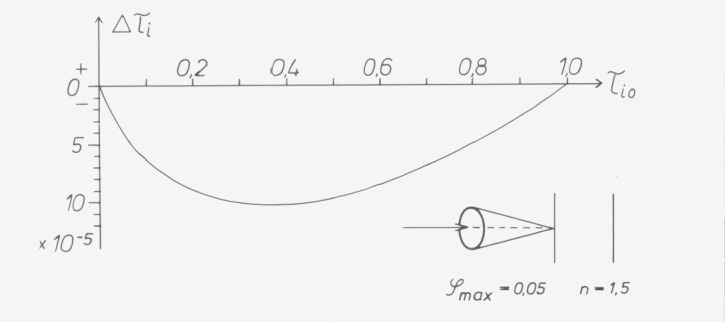
Apparent nonlinearity due to oblique incidence calculated from eqs (12) and (13) for a circular cone with the aperture angle ϕ_max_=0.05 and n=1.5.

The curves in figure 18 are calculated with *φ*_max_=0.05 and *n*=1.5 to illustrate the eqs (12) and (13). The errors have been calculated by Hansen and Mohr for two apertures of finite size [21].

If the sample is tilted by the angle Ψ towards the optic axis, which may be necessary because of multiple reflections, Mielenz [22] stated for a point source and a square aperture the equation

$$\Delta \tau_{i}=-\frac{1}{3n^{2}}\cdot \tau_{i0}\cdot \ln\;(1/\tau_{i0})\cdot (\varphi_{\max}^{2}+1.5\Psi^{2})$$ (17)

The reflection error is caused by oblique rays which are reflected differently than normal rays. The reflection of the component polarized parallel with the plane of incidence decreases, while the reflection of the perpendicularly polarized component increases. The effects are oppositely equal for small angles, which means that they cancel when unpolarized light or a symmetrical cone is used. Mielenz indicates the following equation [22] for the tilted sample

$$\Delta \tau =\pm 4\tau \cdot \frac{r}{n}\cdot \Psi^{2}.$$ (18)

### C. Influence of Bandwidth

If the bandwidth used for measurement is not small enough to assume that the transmittance of the material within the bandwidth is constant, the spectrophotometer will supply an average value of the transmittance within the bandwidth, which is based on the internal transmittance

$${\bar{\tau }}_{i}=\int g(\lambda )\cdot \tau_{i}(\lambda )\cdot d\lambda$$ (19)

where *g*(*λ*) is the weight with which the individual wavelengths contribute to the total signal. If only one sample is measured, the conditions are fully described by this equation. However, the situation is different if a thicker sample of the same material is measured, which has not only a different internal transmittance but also another wavelength dependency. Terming the absorption coefficient at a mean wavelength *a*_0_ and that at a different wavelength *α*(λ), and the difference of the two values Δ*α*(λ), and defining

$$\alpha (\lambda )=\Delta a(\lambda )/a_{0},$$ (20)

it follows for the average value of the internal transmittance that

$${\bar{\tau }}_{i}=\int g(\lambda )\cdot e^{\left(\ln\;\tau_{t0})[1+\alpha (\lambda )\right]}\cdot d\lambda =\tau_{i0}[1-\ln\;(1/\tau_{i0})\cdot \int g(\lambda )\cdot \alpha (\lambda )\cdot d\lambda ].$$ (21)

This equation is similar to equation (11) in the preceding paragraph, and shows the same dependence on transmittance. If non-neutral standards of the same material but with different thicknesses are measured in photometers with different bandwidths, differences are liable to occur between the measurements in accordance with figure 18.

### D. Interreflections

**Figure 19:**
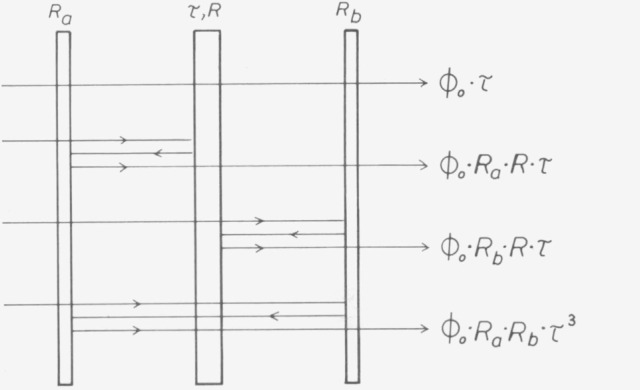
Interreflections in a transmission photometer. *R*_a_ is the reflection coefficient for all optical surfaces before the sample for light incident from the sample side, *R*_b_ that for all surfaces behind the sample. *R* is the reflection coefficient, *τ* the transmittance of the total sample. *ϕ*_0_ is the radiant power flowing directly from source to detector (without interreflections).

Every photometer contains reflecting surfaces before and after the sample. Even if attempts are made to avoid such surfaces, which will be discussed further below, light source and detector themselves will become effective as surfaces of this kind. In commercial photometers the sample is generally arranged between plane or convex windows. The radiant flux will then not simply pass through the sample, but there are interreflected portions, as shown in figure 19, For the radiant flux passing through a sample with the transmittance η and the reflectance *R*_1_ (reflectance of the entire sample, both front and rear surface) it follows that

$$\Phi =\Phi_{0}\cdot \tau_{1}[1+R_{1}\cdot (R_{a}+R_{b})+R_{a}\cdot R_{b}\cdot {\tau_{1}}^{2}].$$ (22)

where *ϕ*_0_ is the radiant flux passing without reflection, and *R_a_, R_b_* are the reflectances at the surfaces in the photometer. Consequently the radiant flux

$$\phi =\phi_{0}[1+R_{a}R_{b}]$$ (23)

passes through the empty photometer. If a second sample (τ_2_, R_2_) is measured relative to the first sample (τ_1_, *R*_1_), the ratio of the radiant fluxes is

$$\phi_{2}/\phi_{1}=\tau_{1}\tau_{2}[1-(R_{1}-R_{2})\cdot (R_{a}+R_{b})-R_{a}\cdot R_{b}\cdot ({\tau_{1}}^{2}-{\tau_{2}}^{2})].$$ (24)

If, however, the second sample is measured in the empty instrument, it follows that (*τ*_1_=l, *R*_1_=0)

$$\phi_{2}/\phi_{1}=\tau_{2}[1+R_{2}\cdot (R_{a}+R_{b})-R_{a}R_{b}(1-{\tau_{2}}^{2})].$$ (25)

In all these equations *R, R*_1_, *R*_2_, *R_a_* and *R_b_* are treated as small quantities; powers higher than *R^2^* are neglected.

Due to these reflections the ratio of the radiant fluxes deviates from the value of the actual transmittance. Part of the reflections can be rendered ineffective by tilting the sample. The errors caused by the tilt must then, of course, be considered. Apart from the errors mentioned in section IV–B, these are the influences of a lateral displacement of the light beam. Mielenz [22] gave a good example, and subsequently Mielenz and Mavrodineanu [23] developed a method to determine (*R_a_+R_b_*) by sample tilting.

However, tilting the sample affects only the term (*R_a_+R_b_*), and does not permit the measurement of *R_a_·R_b_.* An upper limit for this term may be estimated from

$$R_{a}\cdot R_{b}\leq \frac{1}{4}{\left(R_{a}+R_{b}\right)}^{2}.$$ (26)

For direct evaluation of *R_a_·R_b_* the following procedure is suggested here: a sample of approx. 58 percent transmittance (the function *τ*(1−*τ*^2^) has its maximum at $\tau_{M}=1/\sqrt{3}=0.577$) is measured in the photometer under test. It is tilted so that the reflections by the sample do not impair the result, i.e., the term (*R_a_+R_b_*) is eliminated. Two weakly absorbing plates with a transmittance *τ_p_* of approx. 76 percent each are tilted opposite to each other and are placed before and after the sample so that their reflections are excluded, and that they do not cause interreflections with the tilted sample. The opposite inclination cancels possible beam shifts. For the first measured ratio of the radiant fluxes it follows that

ϕ2/ϕ1=τM⋅[1−Ra⋅Rb⋅(1−τM2)], (27)

while the second measurement yields the following radiant fluxes

$$\phi_{3}=\phi_{0}\cdot {\tau_{p}}^{2}[1+{\tau_{p}}^{4}\cdot R_{a}\cdot R_{b}],$$ (28)

$$\phi_{4}=\phi_{0}\cdot {\tau_{p}}^{2}\cdot \tau_{M}[1+{\tau_{M}}^{2}\cdot {\tau_{p}}^{4}\cdot R_{a}\cdot R_{b}].$$ (29)

The ratio of these is

$$\phi_{4}/\phi_{3}=\tau_{M}[1-{\tau_{p}}^{4}\cdot R_{a}R_{b}\cdot (1-{\tau_{M}}^{2})]$$ (30)

and consequently the difference of these ratios is

$$\phi_{4}/\phi_{3}-\phi_{2}/\phi_{1}=\tau_{M}\cdot (1-{\tau_{M}}^{2})(1-{\tau_{p}}^{4})\cdot R_{a}\cdot R_{b}.$$ (31)

*R_a_R_b_* can be calculated from this equation. The reason for the proposed transmittance of 76 percent of the auxiliary plates is the difference 1−*τ_p_*^4^ in eq (31) and the decrease by *τ_p_*^2^ of the total radiant flux. The function *τ*^2^ (1 − *τ*^4^) has its maximum near

$$\tau_{p}=1/\sqrt[{4}]{3}=0.76.$$

The user of a commercial photometer cannot carry out such tests. He cannot work in the cell compartment without windows because of the danger of damage to optical surfaces by spilled chemicals or vapors. If the manufacturer has not eliminated the reflections, the user has to take the instrument as it is. Most spectrophotometers, however, are used to measure solutions. A cell with solution is measured relative to another one containing the solvent. Equation (24) states the “transmittance of the solution relative to the solvent.” The errors tend to cancel, though not completely. Actually the user wants to determine the internal transmittance. If the solvent does not absorb, it follows from the above mentioned definition of true transmittance (equation 5 or 6) that

$$\tau_{2}/\tau_{1}=\tau_{i}[1-r^{2}\cdot (1-{\tau_{i}}^{2})].$$ (32)

For the reflection of the solvent

$$R_{1}=r[1+{\left(1-r\right)}^{2}]$$ (33)

and the reflection of the solution

$$R_{2}=r[1+{\left(1-r\right)}^{2}\cdot {\tau_{i}}^{2}].$$ (34)

If these terms are entered in equation (24) it follows that

$$\phi_{2}/\phi_{1}=\tau_{i}\cdot [1-(1-{\tau_{i}}^{2})\cdot \{r^{2}+(R_{a}+R_{b})\cdot r\cdot R_{a}+R_{b}\}]$$ (35)

The user calculates with the simple formula *ϕ*_2_/*ϕ*_1_*=τ_i_.* Due to the multiple reflections in the instrument (provided they have not been eliminated by the manufacturer) he will experience an apparent nonlinearity according to eq (35) of

$$\Delta \tau_{i}=-\tau_{i}\cdot (1-{\tau_{i}}^{2})\cdot \{r^{2}+(R_{a}+R_{b})r+R_{a}R_{b}\}.$$ (36)

Though higher, it is of the same kind as that expressed in eq (32) for a reflection-free photometer with the following apparent nonlinearity

$$\Delta \tau_{i}=-\tau_{i}(1-{\tau_{i}}^{2})\cdot r^{2}.$$ (37)

The errors are so small that they are negligible in practice. Only very few cases are known to the author where the reflection within the sample was considered according to eq (32) or (37) for analytical applications. Consequently also the somewhat larger error due to eq (36) may be tolerable.

**Figure 20:**
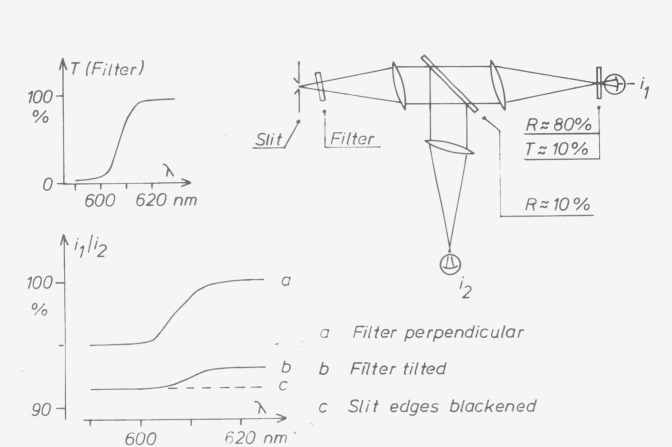
Interreflections in a system with collimated beam (simplified drawing of an arrangement studied by the author for other reasons). The tilted lenses correspond to Mielenz’s proposal of off-axis concave mirrors to eliminate the term *R* (*R*_a_+*R*_b_) in eqs (22) to (25). The interreflective term *R*_a_. *R*_b_ has a very high value in the straight beam from left to right because of the high reflectance of the plate in front of detector 1 (signal i_1_) and a very low one for the beam to detector 2 (signal *i*_2_) because there is no reflecting plate and interreflections are attenuated by the low reflectance of the inclined beam splitting plate. At wavelengths below 600 nm the short cutoff filter suppresses interreflections in both beams, and the variation of the ratio *i*_1_\*i*_2_ with the wavelength shows the influence of interreflections. The filter was at first perpendicular to the beam axis (a), then tilted (b), and finally the small reflecting edges of the slit were carefully blackened. The ratio *i*_1_\*i*_2_ arbitrarily set to 100 percent in one case, shows in curve a below 600 nm the influence of the front surface of the filter and above 620 nm the influence of both surfaces and of the slit edge. Curve b shows above 620 nm the influence of the slit edge alone. In an actual photometer the effects will be smaller depending on the lower reflection values of the detector, but assuming a reflection of 1 percent, the intended accuracy of 10^−4^ will still leave amounts for consideration.

When designing instruments for standards laboratories, multiple reflections should be avoided. Mielenz achieved this by imaging with off-axis parabolic mirrors. This doubtless reduces multiple reflections to values which are neglegible even for most exacting demands with regard to measuring accuracy. However, such an arrangement should always be checked for multiple reflections. Not only must the sample be placed exactly perpendicular to the optic axis, but aberrations must also be kept small and apertures must be blackened. One of these apertures is the monochromator exit slit, and it may be difficult to eliminate reflections from its sharp edges (fig. 20). In Mielenz’s instrument, a blackened monochromator exit aperture was used successfully.

**Figure 21:**
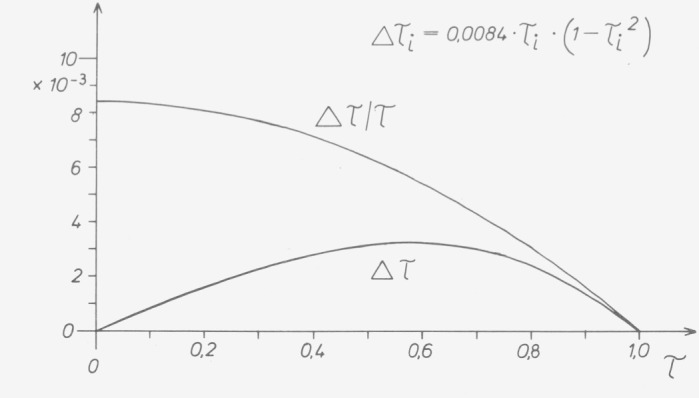
Correction of interreflections and oblique incidence applied to ELKO II photometer. Correction is positive because of the negative error.

**Figure 22:**
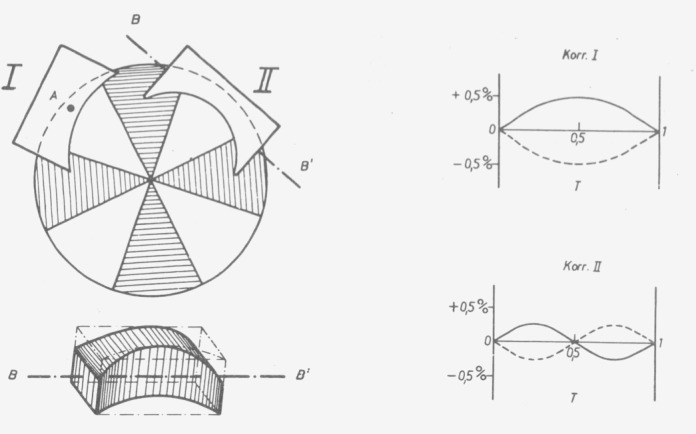
Correctors for the linearity adjustment of the ZEISS ELKO II photometer designed by Hansen [24]. The instrument is operated according to the “optical null” principle with a measuring diaphragm (shown in the drawing) consisting of 4 dark sectors of 45° central angle each, and 4 variable sectors with maximum 45° open angle. Corrector 1 is rotatable around an axis *A* perpendicular to the diaphragm plane, corrector 2 around an axis *B* parallel to the diaphragm plane. Also shown (as curves) are the linearity changes brought about by the correctors.

To avoid reflections the instrument designer can equip the instrument with off-axis mirrors (see above), tilt lenses and windows, or provide them with antireflection coating (which is, however, possible only for a limited spectral range). As far as the linear function of the photometer can be influenced, he can correct the apparent linearity error resulting from eq (35). In 1953 the author did this for ZEISS’s ELKO II photometer.^2^ The apparent errors according to eq (36) were eliminated together with the apparent errors from eq (12) which depend similarly on the transmittance, and possible genuine linearity errors [17]. They were eliminated by so-called correctors described by Hansen [24] (figs. 21, 22). The following numerical value was determined for correction:

$$\Delta \tau_{i}=+0.0084\tau_{i}(1-{\tau_{i}}^{2}).$$

The above considerations reveal a danger in testing photometers with multiple reflections by standards which are calibrated against air. Multiple reflections do not only supply higher absolute values than actually available of the linearity error, but compared with the error involved in the measurement of solutions against solvent this error has the opposite sign (compare eqs 25 and 35). If standards are used which are pairs of equally reflecting, yet differently absorbing substances, the errors caused by multiple reflections when measuring the standards and the samples themselves will be at least approximately equal, and the error caused by a multiple reflection will be eliminated at least approximately by the linearity correction.

### E. Interference

The calculation of multiple reflection given in section IV–D is based on the reflection coefficient of the radiant flux. With strictly collimated and monochromatic light, there will be interferences at the parallel surfaces. The transmittance of such a sample will show periodic maxima and minima as a function of the wavelength. This is well known from IR instrument applications, but is also observed in the visible under special conditions. Mielenz has stated corresponding formulae [22]. These interferences are generally regarded as disturbances in spectrophotometric measurements and are eliminated, if possible. There are three ways to achieve this: sufficiently large bandwidth, varying thickness of the sample, and sufficiently large aperture angles.

If the sample has a thickness *d* and a refractive index *n* the interference maxima at the wavelength λ will be at wavelength intervals of

$$\Delta \lambda_{I}=\lambda^{2}/(2nd).$$ (38)

For a glass sample (*n*=1.5) of 1 mm thickness this yields at 500 nm a distance of 0.08 nm, and at 1000 nm as much as 0.33 nm. To average the interferences the photometer bandwidth Δλ*_h_* should be at least ten times the distance between interference maxima, [28] that is

$$\Delta \lambda_{h}\geq 10\Delta \lambda_{I}=10\lambda^{2}/(2nd)$$ (39)

which means that at 1000 nm the bandwidth must be at least 3 nm for a thickness of 1 mm, or that at 1 nm bandwidth the thickness must be at least 3 mm.

To get from one inference maximum to the next by changing the thickness, the change of thickness must be

Δd=λ/2n. (40)

If again 10 interference maxima (Fizeau fringes) are to be averaged [28], a thickness change of 3.3 *μ*m is necessary at 1000 nm. Provided this is to be below 10^−5^ of the thickness, the sample should be at least 33 cm thick. Even if constancy of the thickness to within only 10^−4^ of its value is required, the required sample thickness would still be 3.3 cm. Interferences can thus not be compensated by intentional variation of the sample thickness over the measuring area unless the accuracy requirements are low.

The same applies to the compensation of interference by the use of larger aperture cones. The relation of oblique beam passage and change in path length has been derived in section IV-B. The same limits as mentioned above are true because, although with oblique rays (Haidinger rings) the change of the transmitted layer is desirable to compensate the interference, it is undesirable for the constancy of absorption. Interferences cannot be effectively compensated by the aperture cone in collimated-beam photometers of standards laboratories, however, the larger cone in commercial photometers causes freedom from interferences.

To calibrate standards of high accuracy, several measurements must be made at intervals of approx. ⅛ of the wavelength difference indicated in eq (38), followed by averaging over one period of the interference, if interferences are detected.

The small increase in transmittance of gray glasses over the years may also be considered as an interference phenomenon. It is due to the formation of surface layers by a kind of aging, which have a reflection-reducing effect. For this reason, gray glasses should not be used alone but in combination with other glass types. It has up to now not been possible to obtain glass of equal refractive index and chemical composition which changes with time in the same manner as gray glasses.

### F. Polarization

As a rule, the light in commercial photometers is partially polarized. The horizontal and vertical cross sections of the light beams being of different shape, this polarization affects the reflection at oblique incidence. If such a photometer is tested with standards having surfaces similar to those of the cells used for measurement, the error caused by polarization is corrected together with the linearity error.

An instrument with rotational symmetry of the beam cross section must be used to calibrate the standards. If this proves impossible, measurements must be made in the two preferred polarization directions.

To avoid systemmatic errors in partially polarized light, the standards should be free from birefringence, strain or optical activity.

### G. Beam Shift Errors

As mentioned before, the sensitivity of all known photomultiplier tubes depends considerably on the position on the cathode and on the direction of incidence. If the beam is shifted when the sample is brought into the beam path, errors occur which differ even with instruments of the same type. Shifts of the light beam on the cathode may be due to wedge errors, tilt errors or focusing errors. This changes the cross section of the light beam at the cathode. If collimated light passes through the sample, the tilt and focusing errors will not shift the beam cross section in the focal plane of the collimator but the direction of the beams. If the light beam falls directly on the detector the cross section on the detector should not be too small. This is the reason for producing an image of the pupil on the detector. However, even with collimated light the sample may cause changes of the pupil image.

The best solution is to eliminate the dependency of the sensitivity on place and direction. This can be achieved with an averaging sphere, which because of its low efficiency has so far only been used in special equipment. Whether or not the progress made in designing averaging spheres [25] will make them suitable for commercial spectrophotometers remains to be settled.

## V. How Can Routine Spectrophotometry Be Improved?

Whoever wants to improve spectrophotometry must know the inherent sources of error. According to the author there are enough means to test the spectral characteristics. But, to test the linearity of the transmittance scale, standards are required which must be issued by a standards laboratory. As far as the author knows there are being offered only two types of standards which are calibrated according to independent and published procedures: the gray glasses and solutions issued by NBS and the gray glasses of ZEISS (see note [26]).

A standards laboratory will be responsible for the increase in accuracy up to a technically feasible limit. Important progress has recently been made in this respect. Yet the errors mentioned at the beginning are about 3 orders of magnitude above the accuracies obtained in standards laboratories. It would be an important step forward if an accuracy of a few tenths of a percent were achieved for routine applications. Standards with transmittances guaranteed to within approx. 0.1 percent will do. They must be easy to handle and to clean and must, of course, be stable. They should also be neutral. Gray glasses meet these specifications for the visible spectral range, but the formation of a surface layer impairs the stability of the values with time. Changes of up to 1 percent of actual transmittance have been observed by us within ten years. If a material of higher stability is not found, it should be tried to calibrate these glasses with reference to a similar absorption-free glass, which would eliminate most of the time-dependency [27]. This would best meet practical requirements, and would ensure the smallest influence by multiple reflections.

Gray glasses cannot be used in the UV. Blackened quartz glass being commercially available, attempts should be made to produce quartz glass which absorbs in the UV almost independent of the wavelength.

Vaccum-deposited, neutrally transmissive metal coatings can be used within a much wider spectral range than glasses. In spite of this the author doubts their usefulness even for moderate accuracy requirements, because they reflect too much light. Even if such filters are tilted (by means of a suitable mount, for instance) to eliminate part of the errors due to multiple reflection, this may cause errors in commercial spectrophotometers, because the reflected light is much stronger than the reflection at glass surfaces; even a reflection on to a black surface may cause measuring errors. Furthermore, these coatings are very sensitive, but in spite of this the author would not recommend cementing with a coverglass, because all cementing agents are known to increase their UV absorption with time.

Solutions, even if they are transported in sealed ampouls, are still problematic with regard to durability, contamination and the cells required for their use. Other means not being available at present, they are to the author’s knowledge the only solution for the UV.

Photometers with fixed cells to measure liquids continuously or in cycles should be tested with reference to gray glasses. These usually can be inserted, because the cells must be removable for cleaning and replacement. Liquids are needed in the visible spectral range to test such photometers only if the cell cuts off the beam path and an additional gray glass cannot be provided.

The improvement of routine spectrophotometry is more a problem of instruction of the user and provision of suitable equipment than of improving the accuracy in the standards laboratories.

## Figures and Tables

**Table I:** Best, medium and worst values selected from table I group A of Beeler and Lancaster [1] C.V. = Coefficient of Variation St.D. = Standard Deviation *T* Δ*T/T*=2.30·Δ*A* and Δ*T* have been calculated from *A* and Δ*A/A* given by B. and L.

Solution p. = potassium	Conc. mg/1	Wav. nm	Δ*A/A* C.V.%	*A*	*T %*	Δ*T/T* C.V.%	Δ*T* St.D.%
							
Acid p. dichromate	20	380	11.1	0.109	77.8	2.79	2.17
Alkaline p. Chromate	40	300	15.1	.151	70.9	5.25	3.72
Alkaline p. Chromate	40	340	9.2	.318	48.3	6.74	3.25
Acid p. dichromate	60	328	5.0	.432	38.0	4.97	1.88
Acid p. dichromate	100	366	5.8	.855	14.0	11.42	1.59
Acid p. dichromate	100	240	2.8	1.262	5.47	8.14	0.45

**Table II:** Emission lines of hydrogen and deuterium

	H	D
		
*α*	656.285 nm	656.100 nm
*β*	486.133	485.999

**Table III:** Variation of photocathode sensitivity with temperature [8]

Photocathode	Blue	Red
		
Ag O Cs	−0.24 %/°C	−0.14 %/°C
Sb Cs	−0.48	+ 0.64
Multialkali	−0.22	−0.24

**Table IV:** Shot noise influence on precision of transmission measurements if the cone angle *φ*_max_ is limited by acceptable path length error *∊.* Calculated from eq (9) with Δλ = 1 nm, *q* = 0.1, *A* = 0.196 cm^2^ (beam diameter 0.5 cm), *n* = 1.5 for λ = 400 nm with *τ* = 0.1 and *L*_λ_ = 2 mW/cm^2^ sr nm (Tungsten coil) and for λ=200 nm with *τ* = 0.01 and *L*_λ_ = 0.8 mW/cm^2^ sr nm (Deuterium lamp). If the measuring time is assumed to be 1 s for 100 percent transmittance or 10 s for 10 percent transmittance, relative precision remains the same.

*∊*	*φ*_max_ rad	*φ*_max_ deg	Δ*N/N* (400 nm)	Δ*N/N* (200 nm)
				
10^−3^	6.71 10^−2^	3.84	3.0 10^−6^	2.1 10^−5^
10^−4^	2.12 10^−2^	1.215	0.95 10^−5^	6.7 10^−5^
10^−5^	6.71 10^−3^	0.384	3.0 10^−5^	2.1 10^−4^

## Footnotes

1 Figures in brackets indicate the literature references at the end of this paper.

2 In order to describe materials and experimental procedures adequately, it was occasionally necessary to identify a commercial product by the manufacturer’s name. In no instances does such identification imply endorsement by the National Bureau of Standards.
